# Lung Epithelial TRPA1 Transduces the Extracellular ROS into Transcriptional Regulation of Lung Inflammation Induced by Cigarette Smoke: The Role of Influxed Ca^**2+**^

**DOI:** 10.1155/2015/148367

**Published:** 2015-10-04

**Authors:** An-Hsuan Lin, Meng-Han Liu, Hsin-Kuo Ko, Diahn-Warng Perng, Tzong-Shyuan Lee, Yu Ru Kou

**Affiliations:** ^1^Department of Physiology, School of Medicine, National Yang-Ming University, Taipei 11221, Taiwan; ^2^Department of Chest Medicine, Taipei Veterans General Hospital, Taipei 11217, Taiwan

## Abstract

The mechanism underlying the inflammatory role of TRPA1 in lung epithelial cells (LECs) remains unclear. Here, we show that cigarette smoke extract (CSE) sequentially induced several events in LECs. The Ca^2+^ influx was prevented by decreasing extracellular reactive oxygen species (ROS) with the scavenger N-acetyl-cysteine, removing extracellular Ca^2+^ with the chelator EGTA, or treating with the TRPA1 antagonist HC030031. NADPH oxidase activation was abolished by its inhibitor apocynin, EGTA, or HC030031. The increased intracellular ROS was halted by apocynin, N-acetyl-cysteine, or HC030031. The activation of the MAPKs/NF-*κ*B signaling was suppressed by EGTA, N-acetyl-cysteine, or HC030031. IL-8 induction was inhibited by HC030031 or TRPA1 siRNA. Additionally, chronic cigarette smoke (CS) exposure in wild-type mice induced TRPA1 expression in LECs and lung tissues. In CS-exposure *trpa1* ^−/−^ mice, the increased BALF level of ROS was similar to that of CS-exposure wild-type mice; yet lung inflammation was lessened. Thus, in LECs, CSE may initially increase extracellular ROS, which activate TRPA1 leading to an increase in Ca^2+^ influx. The increased intracellular Ca^2+^ contributes to activation of NADPH oxidase, resulting in increased intracellular ROS, which activate the MAPKs/NF-*κ*B signaling leading to IL-8 induction. This mechanism may possibly be at work in mice chronically exposed to CS.

## 1. Introduction

Inhaled cigarette smoke (CS) causes persistent lung inflammation that leads to the development of chronic obstructive pulmonary disease (COPD) in smokers [1]. CS-induced lung inflammation is well recognized as being regulated by a complex mechanism that involves various types of cells and a range of inflammatory mediators [1, 2]. For example, the chemokine interleukin-8 (IL-8), when released from lung epithelial cells, plays a vital role in the regulation of lung inflammation [3–7]. This is due to the fact that the lung epithelium is a target for direct insult by CS and that chemokines are able to promote inflammatory cell recruitment into the lungs [2, 5]. The induction of inflammatory mediators in lung epithelial cells [3–7] or other types of lung cells [8] by CS is mainly regulated by redox-sensitive signaling pathways. CS is a potent oxidant that has the ability to initially cause increases in reactive oxygen species (ROS) in extracellular fluid [7, 9] and in bronchoalveolar lavage fluid (BALF) [7, 10]. Subsequently, via activation of NADPH oxidase, CS increases intracellular ROS levels in lung epithelial cells [6, 7] and other types of lung cells [11–14]. NADPH oxidase is known to be the primary enzyme system able to generate ROS in mammalian cells [15]. This increase in intracellular ROS in epithelial cells is known to activate several ROS-sensitive signaling pathways, including the mitogen-activated protein kinases (MAPKs) and various downstream transcriptional factors, such as nuclear factor-*κ*B (NF-*κ*B); these effects then ultimately promote inflammatory gene expression [3, 4, 6–8]. However, the role of increased extracellular ROS in the CS-induced regulation of lung inflammation has remained largely unclear up to the present.

Transient receptor potential ankyrin 1 (TRPA1) is a type of nonselective transmembrane cation channel that is mainly involved in Ca^2+^ permeability [16]. TRPA1 was originally thought to be predominately expressed in primary sensory neurons [16]. Lung neuronal TRPA1 has been suggested to act as an oxidant sensor allowing detection of pulmonary ROS that is induced by oxidants such as CS or H_2_O_2_; this results in the elicitation of neural impulses in lung sensory fibers [17–19]. However, recent studies have demonstrated that TRPA1 is also expressed in various types of nonneuronal cells, including lung epithelial cells [17, 20–22]. Indeed, stimulation of lung epithelial cells by TRPA1 agonists or by CS extract (CSE) increases the production of IL-8 and this effect is able to be attenuated by TRPA1 antagonists [21, 22]. Additionally, when exposed to CS for 3 days, TRPA1 knockout (*trpa1* ^−/−^) mice display a lower level of keratinocyte chemoattractant (an IL-8 analogue) in BALF, as compared to wild-type mice [22]. These observations suggest that nonneuronal lung TRPA1 plays an important role in the CS-induced production of inflammatory mediators. Nevertheless, how nonneuronal TRPA1 in the lung participates in the CS-induced transcriptional regulation of these mediators has remained elusive.

The aims in this study were, firstly, to determine whether lung epithelial TRPA1 is able to be activated when there is an increase in extracellular ROS; this was done using an* in vitro* model involving primary human bronchial epithelial cells (HBECs) and exposure of these to CSE [6, 7]. Secondly, we wished to investigate how epithelial TRPA1 may act as a crucial regulator in the activation of NADPH oxidase and thus promote the subsequent transcriptional regulation of IL-8 in HBECs. Thirdly, we wanted to assess the importance of lung epithelial TRPA1 to CS-induced lung inflammation using a murine model that consisted of chronic CS exposure for 4 weeks [6, 7, 23].

## 2. Materials and Methods

### 2.1. Reagents

Antibodies (Abs) and ELISA kits for the detection of IL-8 and MIP-2 were purchased from R&D Systems (Minneapolis, MN, USA). Rabbit Ab against c-Jun N-terminal kinases (JNK) was obtained from Cell Signaling (Beverly, MA, USA). Mouse Ab against phospho-JNK was purchased from BD (San Jose, CA, USA). Abs against extracellular signal-regulated kinase (ERK), phospho-ERK, and p65 were obtained from Santa Cruz Biotechnology (Santa Cruz, CA, USA). Mouse Abs against *α*-tubulin, HC030031, ethylene glycol tetraacetic acid (EGTA), N-acetyl-cysteine, and apocynin were purchased from Sigma-Aldrich (St. Louis, MO, USA). Mouse Ab against TRPA1 was obtained from Abcam (Cambridge, MA, USA) or from Calbiochem (San Diego, CA, USA). Mouse Ab against histone H1 was purchased from Millipore (Bedford, MA, USA). Mouse Ab against 4-hydroxynonenal (4-HNE) was obtained from Abcam (Cambridge, MA, USA). The Screen Quest Fluo-8 Medium Removal Calcium Assay Kit was purchased from AAT Bioquest (Sunnyvale, CA, USA). The EnzyChrom NADP^+^/NADPH assay kit was obtained from BioAssay Systems (Hayward, CA, USA). The membrane-permeable probes hydroethidine (HE) and 2′,7′-dichlorofluorescin diacetate (DCFH-DA) were purchased from Molecular Probes (Eugene, OR, USA). Scramble and TRPA1 siRNAs were obtained from Ambion (Austin, TX, USA). INTERFERin siRNA transfection reagent was purchased from Polyplus (New York, NY, USA).

### 2.2. Preparation of CSE

CSE was freshly prepared on the day of the experiment as previously described [6, 7] with some modifications. In brief, 1000 mL of the smoke generated from two burning cigarettes (Marlboro Red Label) without filters was sucked at a constant flow rate (8 mL/s) into a syringe and then bubbled into a tube containing 20 mL serum-free medium. The CSE solution was sterilized using a 0.22-*μ*m filter (Millipore, Bedford, MA) and the pH was adjusted to 7.4. The optical density of the CSE solution was determined by measuring the absorbance at 302 nm [24] or 320 nm [25], which, in reality, was found to show little difference between different preparations. This CSE solution was considered 100% CSE and was further diluted with serum-free medium to the desired concentrations that were then used to treat HBECs for different durations of time.

### 2.3. Cell Culture

HBECs (Cascade Biologics, Portland, OR, USA) were cultured in epithelial cell growth medium (medium 200; Cascade Biologics, USA) containing 10% fetal bovine serum (FBS), 1X low serum growth supplement, 100 U/mL penicillin, 100 *μ*g/mL streptomycin, and 0.25 *μ*g/mL amphotericin B (Biological Industries, Kibbutz Beit Haemek, Israel) at 37°C in an incubator with 5% CO_2_.

### 2.4. Measurement of Intracellular Ca^2+^ Levels

Intracellular Ca^2+^ levels were determined using a Screen Quest Fluo-8 Medium Removal Calcium Assay Kit according to the manufacturer's instructions.

### 2.5. Determination of NADPH Oxidase Activity

The activity of NADPH oxidase was examined using an EnzyChrom NADP^+^/NADPH assay kit according to the manufacturer's instructions. This assay kit measures the change in NADP^+^/NADPH ratio in cellular lysate samples and reflects the relative NADPH oxidase activity in the samples tested.

### 2.6. Western Blot Analysis

Aliquots of cell lysates or tissue lysates were separated by 8–12% SDS-PAGE and then transblotted onto Immobilon-P membrane (Millipore). After being blocked with 5% skim milk, the blots were incubated with various primary antibodies and then appropriate secondary antibodies. The specific protein bands were detected using an enhanced chemiluminescence kit (PerkinElmer), which was followed by the quantification using ImageQuant 5.2 software (Healthcare Bio-Sciences, Philadelphia, PA, USA).

### 2.7. Reverse Transcription-Polymerase Chain Reaction (RT-PCR)

Total RNA was isolated from cells using Tri reagent and converted into cDNA using reverse transcriptase (Biolabs, Ipswich, New England) with oligo-dT as the primer. The cDNAs thus produced were then used as templates for the semiquantitative PCR. PCR was performed in a DNA Thermal Cycler (Biometra Tpersonal, Horsham, PA, USA) using the following program: 94°C for 2 min, followed by 35 cycles of 94°C for 30 sec, 58°C for 30 sec, and 72°C for 1 min and then a final single cycle of 72°C for 10 min. The nucleotide sequences of the primers were as follows: TRPA1 sense: 5′-TCACCATGAGCTAGCAGACTATTTAATTT-3′, antisense: 5′-ATGAGAGCGTCCTTCAGAATCG-3′ and GAPDH sense: 5′-TGT TCC AGT ATG ACT CCA CTC-3′, antisense: 5′-TCC ACC ACC CTG TTG CTG TA-3′.

### 2.8. Small Interfering RNA (siRNA) Transfection

HBECs were transfected with scramble or TRPA1 siRNA using INTERFERin siRNA transfection reagent for 24 hours. The TRPA1 siRNA consisted of a mixture of sc-44780A, sc-44780B, and sc-44780C. The nucleotide target sequences of sc-44780A were sense, GCUAAGCCAUGUAAAUCAAtt, and antisense, UUGAUUUACAUGGCUUAGCtt. The nucleotide target sequences of sc-44780B were sense, CUGACAUAGUCCUGAACAAtt, and antisense, UUGUUCAGGACUAUGUCAGtt. The nucleotide target sequences of sc-44780C were sense, CCAUUCUCGUUGUCAAUAUtt, and antisense, AUAUUGACAACGAGAAUGGtt.

### 2.9. Murine Model of Chronic CS Exposure

All animal experiments were approved by the Institutional Animal Care and Use Committee of the National Yang-Ming University (Approval Number: 991220). The murine model of chronic CS exposure has been described in detail previously [6, 7, 23]. Briefly, male* trpa1* ^−/−^ mice (Jackson Laboratory, Maine, USA) and wild-type C57BL/6J mice (National Laboratory Animal Center, Taipei, Taiwan) at the age of 8 weeks were randomly divided into four groups for exposure to air or CS; the groups were, namely, air-wild-type, air-*trpa1* ^−/−^, CS-wild-type, and CS-*trpa1* ^−/−^. At each CS exposure, the mice were placed in an exposure chamber and 750 mL of fresh CS generated from 1.5 cigarettes (Marlboro Red Label; 10.8 mg nicotine and 10.0 mg tar per cigarette) was delivered to the chamber. The CS passed out of the chamber via four exhaust holes (1 cm) on the side panels. During the exposure, the mice were conscious and breathing spontaneously and the treatment in the chamber lasted for 10 min. After exposure, the mice were transferred to a new cage and allowed to inspire air normally. The mice were exposed at 10:00 and 16:00 each day for 4 weeks. The control animals underwent identical procedures in another chamber but were only exposed to air. For each CS exposure, the particle concentration inside the exposure chamber was about 625 mg/m^3^ initially but decreased overtime due to the fact that the CS passed out of the chamber via the exhaust holes [7]. The HbCO levels immediately after the 10-minute exposure protocol for air-exposure and CS-exposure mice were 0.42% and 31.48% (*n* = 6), respectively [7].

### 2.10. *En Face* Immunostaining of the Whole Lung

Male* trpa1* ^−/−^ mice and wild-type C57BL/6J mice at the age of 8 weeks were euthanized with CO_2_ and a middle thoracotomy was performed. The lungs were then infused with 4% paraformaldehyde (0.8 mL) overnight. After infusion with 3% H_2_O_2_ and being blocked with bovine serum albumin, the lungs were infused with IgG or TRPA1 Ab overnight at 4°C and then with HRP-conjugated corresponding secondary Ab for 1 hour. The antigenic sites were visualized by the addition of DAB. The lung was then soaked in xylene and its image was photographed by a digital camera (Sanyo, Osaka, Japan).

### 2.11. Preparation of BALF and Lung Tissues

At the end of each experiment, the mice were euthanized with CO_2_ and a middle thoracotomy was performed. The left lung was ligated and the right lung was lavaged four times with 0.6 mL of warm PBS containing a complete protease inhibitor cocktail (Roche Diagnostics, Mannheim, Germany). The BALF samples were then centrifuged at 350 ×g for 5 min at 4°C, and the supernatant of the first lavage fluid was stored at −80°C for later analysis of total protein using Bio-Rad protein assay reagent (Bio-Rad Laboratories, Hercules, CA, USA). The cell pellets of the BALF samples were resuspended in PBS for cell counting. Furthermore, the right lung was then stored at −80°C for subsequent analysis. The left lung was fixed with 4% paraformaldehyde and embedded in paraffin.

### 2.12. Histological and Immunohistochemical Assessments

Formalin-fixed, paraffin-embedded tissue blocks were cut into 8-*μ*m sections. The sections were deparaffinized, rehydrated, and finally stained with hematoxylin and eosin (H&E) staining; they were then viewed under a microscope (Motic TYPE 102M, Xiamen, China). The histological assessments were conducted by a pathologist who was blinded to the data. Each histological characteristic was scored on a scale of 0-normal to 5-maximal. Lung inflammatory score was categorized according to the sum of the score of infiltration cell numbers and damage levels, including thickening of alveolar walls and epithelium, as well as increases in peribronchial and perivascular cuff area. For immunohistochemical assessment, sections were deparaffinized, rehydrated, and then covered with 3% H_2_O_2_ for 10 min. After being blocked with bovine serum albumin, each slide was first incubated with primary antibodies for 1 hour at 37°C, followed by the corresponding secondary antibodies for an additional hour. The color of all of the sections was developed with 0.1% diaminobenzidine (DAB) and then the sections were counterstained with hematoxylin; this was followed by examination under a microscope. The detected signal was digitally captured using an image analysis system (Image-Pro Plus 4.5, Media Cybernetics, Bethesda, MD, USA) as described previously [7, 26]. The intensity of the immunoreaction developed within epithelium was then assessed densitometrically. Ten epithelial cells were analyzed for each section and three different sections were analyzed for each animal. The data were averaged for each animal and are expressed in arbitrary units.

### 2.13. Measurement of an Oxidative Stress Biomarker

Levels of 4-HNE modified proteins, a product of lipid peroxidation, in lung tissues were measured and this served as a biomarker of oxidative stress as described previously [27].

### 2.14. Measurement of Extracellular and Intracellular ROS Levels

The membrane-permeable probes HE and DCFH-DA were used to assess levels of ROS using methods that have been described previously [28, 29]. For the* in vitro* study, HBECs were incubated in culture medium containing 10 *μ*M HE at 37°C for 30 min. After stimulation with CSE for the desired time, the culture medium was removed for the measurement of extracellular ROS levels. The cells were washed and detached with trypsin/EDTA to allow the measurement of intracellular ROS levels. For the* in vivo* study, the supernatant of the first BALF sampled from all study groups was incubated with 10 *μ*M DCFH-DA at 37°C for 15 min. The fluorescence intensities of the culture medium, cells, and BALF samples were then analyzed using a multilabel counter (PerkinElmer, Waltham, MA, USA). Images of the cells were also obtained by examining them using a Nikon TE2000-U florescence microscope (Tokyo, Japan).

### 2.15. Determining the Concentration of Macrophage Inflammatory Protein 2 (MIP-2)

The concentrations of MIP-2 in BALF and in lung tissue were measured using an ELISA kit according to the manufacturer's instructions.

### 2.16. Statistical Analysis

The results are presented as mean ± SEM. Statistical evaluations involved one-way ANOVA followed by Dunnett's test or Fisher's least significant difference procedure for multiple comparisons as appropriate. Differences were considered statistically significant at *p* < 0.05.

## 3. Results

### 3.1. CSE Increases the Expression of TRPA1 in HBECs

**Figure 1:**
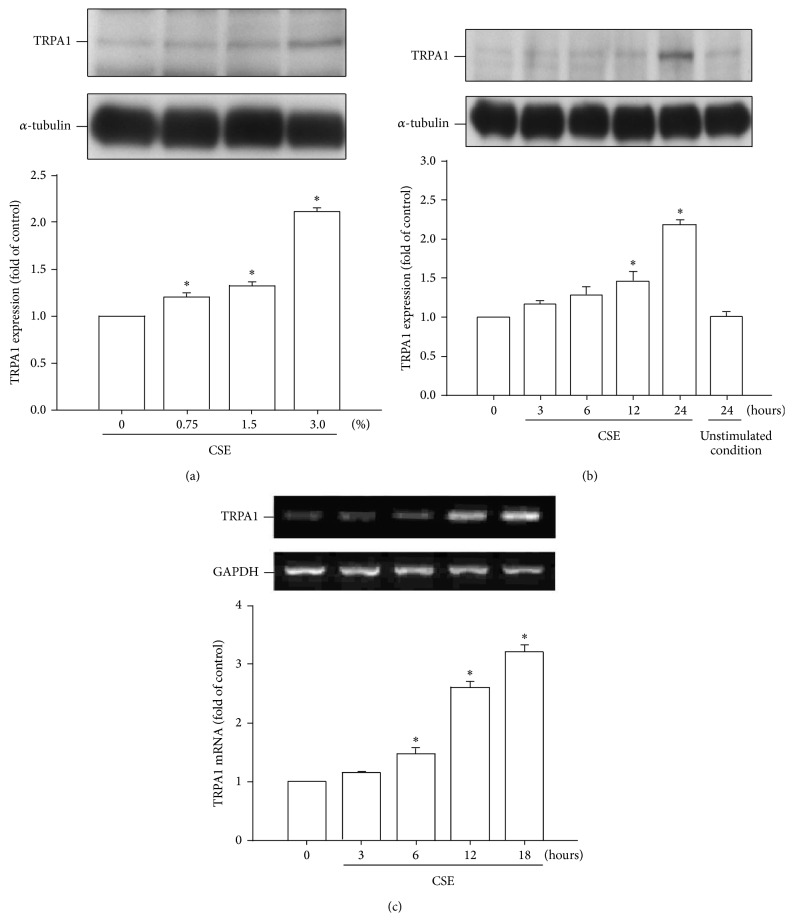
Cigarette smoke extract (CSE) concentration-dependently and time-dependently increases TRPA1 expression in human bronchial epithelial cells (HBECs). (a) Cells were exposed to 0–3% CSE for 24 hours. (b) Cells were incubated with medium alone at time 0 and for 24 hours or exposed to 3% CSE for the indicated times. (c) Cells were exposed to medium alone or exposed to 3% CSE for indicated times. Protein (a and b) and mRNA (c) levels in the cell lysates were analyzed by Western blotting and RT-PCR, respectively. The data in each group are mean ± SEM from five independent experiments. ^∗^ *p* < 0.05 versus the control or versus time zero.

We found that exposure of HBECs to various concentrations (0, 0.75, 1.5, and 3%) of CSE for 24 hours increased the protein level of TRPA1 in a concentration-dependent manner (Figure 1(a)). In addition, exposure of HBECs to 3% CSE for up to 24 hours time-dependently increased the protein level of TRPA1 (Figure 1(b)). Furthermore, an elevation in the mRNA level of TRPA1 was also detected at 6–18 hours after CS exposure (Figure 1(c)).

### 3.2. TRPA1 Is Important to the Induction of IL-8 by CSE in HBECs

**Figure 2:**
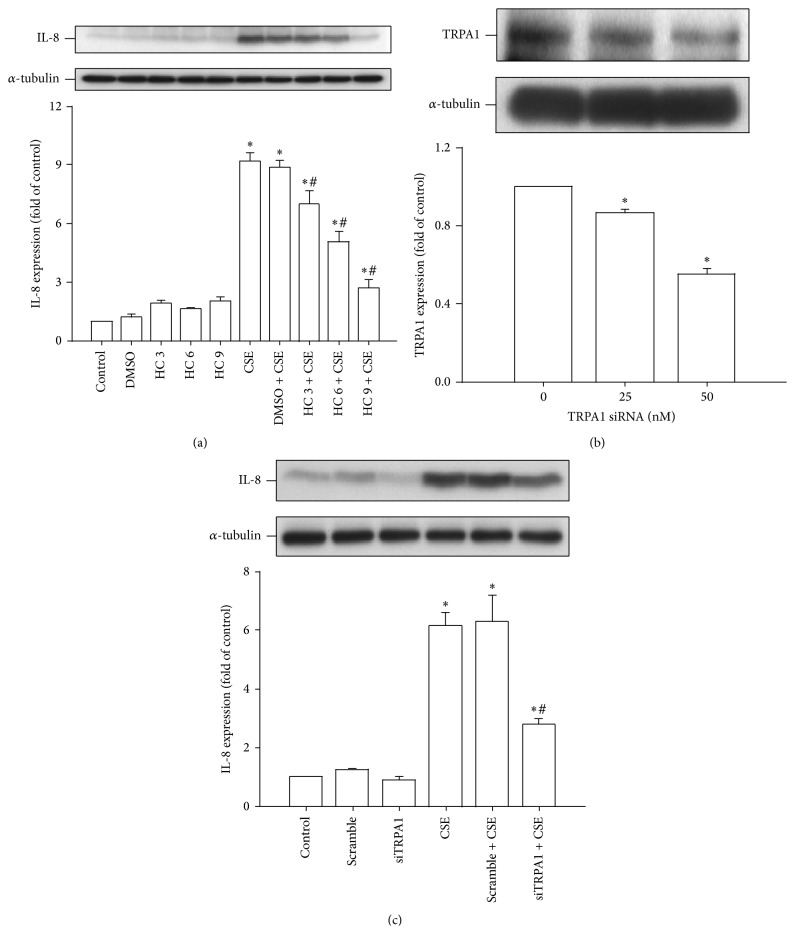
TRPA1 is important for the induction of IL-8 by CSE in HBECs. (a) Cells were incubated with medium alone or exposed to 3% CSE for 24 hours and pretreated with 3–9 *μ*M HC-030031 (HC, a TRPA1 antagonist) or its vehicle DMSO. (b) Cells were incubated with or without two concentrations (25 and 50 nM) of TRPA1 siRNA (siTRPA1) for 24 hours prior to the measurement of TRPA1 expression. (c) Cells were incubated with medium alone or exposed to 3% CSE for 24 hours having been pretreated with 50 nM of either siRNA (siTRPA1) or scramble siRNA. Data in each group are mean ± SEM from five independent experiments. ^∗^ *p* < 0.05 versus the control or versus time zero; ^#^ *p* < 0.05 versus CSE alone.

Next we determined the role of TRPA1 in the induction of IL-8 by CSE. Based upon the concentration-response relationship and time-response relationship reported previously [6, 7], 3% CSE with exposure for 24 hours was chosen as the standard treatment for all subsequent experiments throughout this study. Pretreatment with HC-030031 (a TRPA1 antagonist; 3–9 *μ*M) was found to dose-dependently attenuate the induction of IL-8 by CSE, whereas pretreatment with DMSO (the vehicle) failed to produce such an effect (Figure 2(a)). Additionally, pretreatment with siRNA (25 or 50 nM) that was designed to bring about gene knockdown of TRPA1 significantly reduced the amount of TRPA1 protein present after activation (Figure 2(b)). Furthermore, pretreatment with TRPA1 siRNA (50 nM) also attenuated the induction of IL-8 by CSE, whereas pretreatment with scramble siRNA failed to produce any such effect (Figure 2(c)). These findings suggest that TRPA1 is important to the induction of IL-8 by CSE in HBECs.

### 3.3. CSE Increases the Level of Intracellular Ca^2+^ via a TRPA1-Mediated Ion Influx

**Figure 3:**
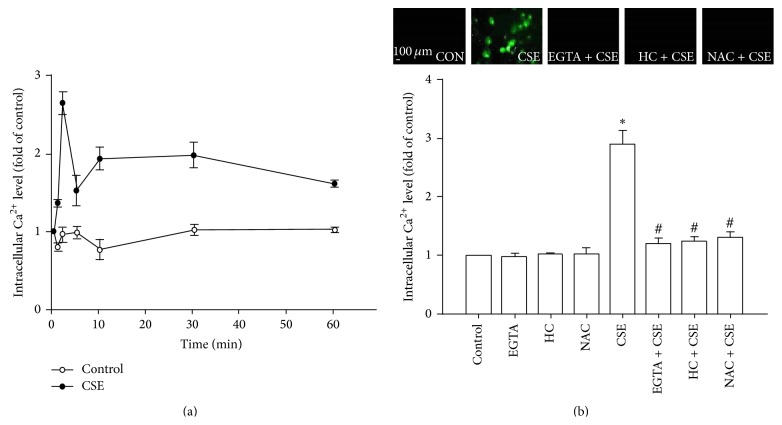
CSE increases the intracellular Ca^2+^ level via TRPA1-mediated ion influx in HBECs. Intracellular Ca^2+^ levels were measured by Fluo-8 fluorescent probe assay. (a) Cells were exposed to medium alone or 3% CSE for 1, 2, 5, 10, 30, and 60 min. (b) Cells were exposed to medium alone or 3% CSE for 2 min and pretreated with EGTA (an extracellular Ca^2+^ chelator; 500 *μ*M), HC-030031 (HC, a TRPA1 antagonist; 9 *μ*M), or N-acetyl-cysteine (NAC, a ROS scavenger; 1 mM). Data in each group are mean ± SEM from five independent experiments. ^∗^ *p* < 0.05 versus the control or time zero; ^#^ *p* < 0.05 versus CSE alone.

After exposure of HBECs to CSE, an increase in the intracellular Ca^2+^ level was found to start at 1 min after treatment initiation, to peak at 2 min after treatment initiation, and to have declined somewhat at 5 min after treatment initiation; nevertheless, at 5 min the level was still higher than the baseline level. This elevation in the intracellular Ca^2+^ level was then maintained until the end of the observation period (60 min) (Figure 3(a)). The increase in the intracellular Ca^2+^ level measured at 2 min after CSE exposure was inhibited by pretreatment with EGTA (an extracellular Ca^2+^ chelator; 500 *μ*M), by pretreatment with N-acetyl-cysteine (a ROS scavenger; 1 mM), or by pretreatment with HC030031 (Figure 3(b)).

### 3.4. CSE-Induced Extracellular ROS Stimulates TRPA1 to Increase Intracellular ROS via the Ca^2+^-Dependent Activation of NADPH Oxidase

**Figure 4:**
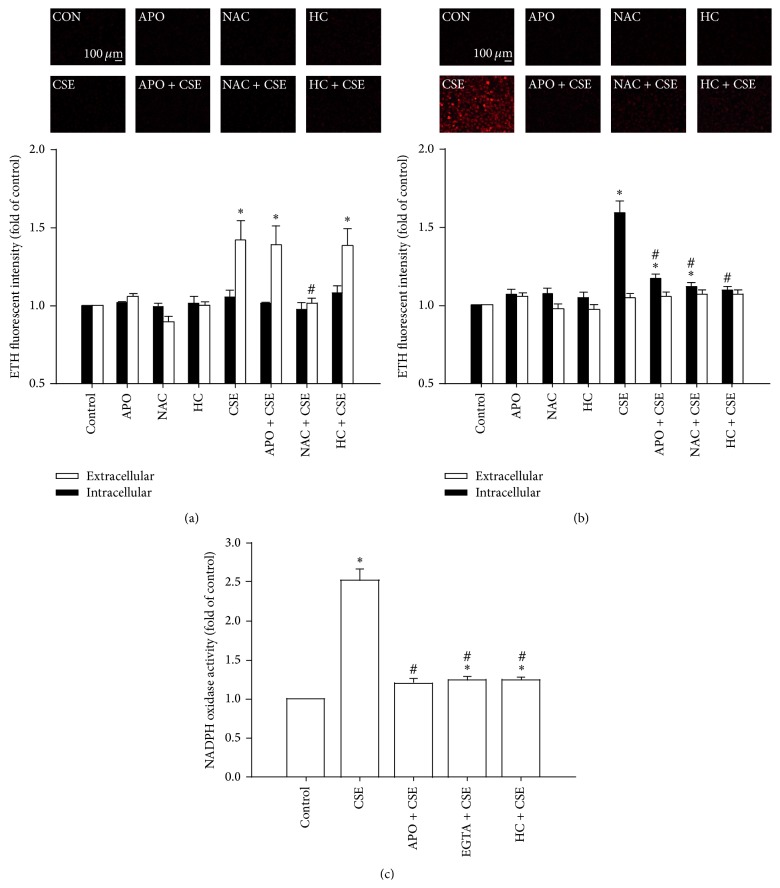
The CSE-induced extracellular ROS stimulate TRPA1 to Ca^2+^-dependently increase intracellular ROS via NADPH oxidase in HBECs. (a–c) Cells were exposed to medium alone or to 3% CSE for 2, 30, and 15 min, respectively, after pretreatment with apocynin (APO; an inhibitor of NADPH oxidase; 150 *μ*M), after pretreatment with N-acetyl-cysteine (NAC, a ROS scavenger; 1 mM), after pretreatment with HC-030031 (HC, a TRPA1 antagonist; 9 *μ*M), or after pretreatment with EGTA (an extracellular Ca^2+^ chelator; 500 *μ*M). Levels of ROS were measured by HE fluorescent probe assay. NADPH oxidase activity was measured by NADP^+^/NADPH assay. Data in each group are mean ± SEM from five independent experiments. ^∗^ *p* < 0.05 versus the control; ^#^ *p* < 0.05 versus CSE alone.

At 2 min after exposure of HBECs to CSE, the extracellular ROS level was significantly increased in the medium; in contrast, at the same time point, the intracellular ROS level had remained unchanged (Figure 4(a)). The CSE-induced increases in extracellular ROS levels in the medium containing HBECs (1.432 ± 0.101-fold of control; *n* = 5) and in the cell-free medium (1.377 ± 0.107-fold of control; *n* = 5) were comparable. This increase in the extracellular ROS level was unaffected by pretreatment with apocynin (an inhibitor of NADPH oxidase; 150 *μ*M) and by pretreatment with HC030031 (9 *μ*M) but was prevented by pretreatment with N-acetyl-cysteine (Figure 4(a)). In contrast to the above results, at 30 min after CSE exposure, it was found that the intracellular ROS level had significantly increased, while the extracellular ROS level had returned to the baseline level (Figure 4(b)). This increase in the intracellular ROS level was prevented by pretreatment with apocynin, by pretreatment with N-acetyl-cysteine, and by pretreatment with HC030031 (Figure 4(b)). Further analysis revealed that, at 15 min after exposure, the presence of CSE had significantly increased the activity of NADPH oxidase and that this was inhibited by pretreatment with apocynin, by pretreatment with EGTA, and by pretreatment with HC030031 (Figure 4(c)).

### 3.5. CSE-Induced Activation of the MAPKs/NF-*κ*B Signaling Is TRPA1-Mediated, Ca^2+^-Dependent, and ROS-Sensitive

**Figure 5:**
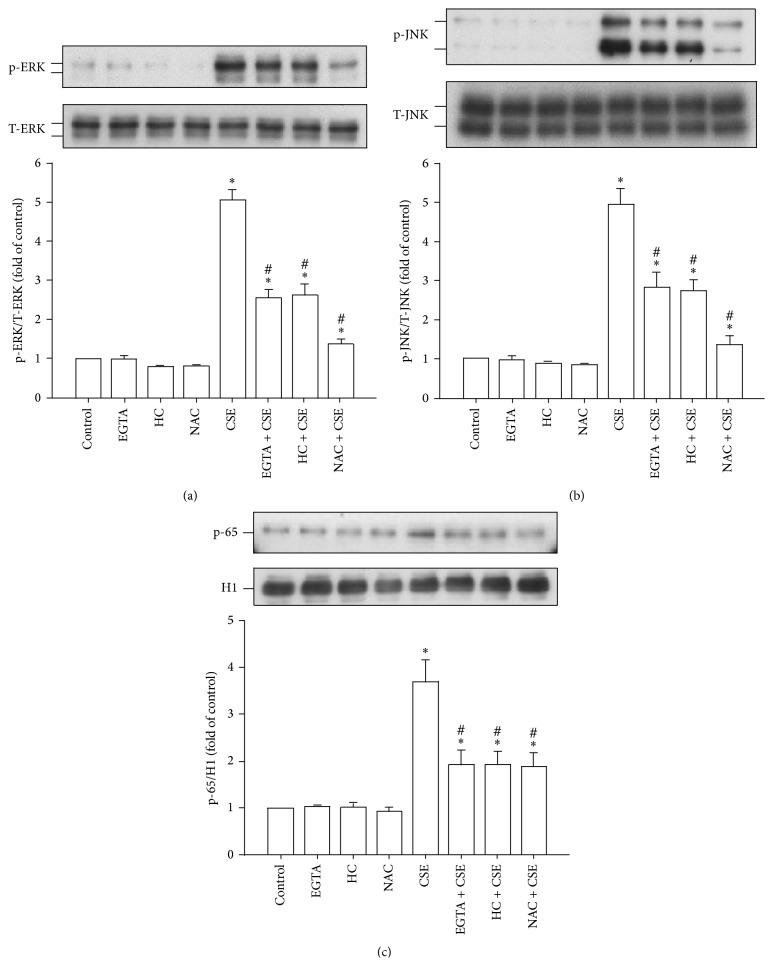
The CSE-induced activation of the ERK/JNK/NF-*κ*B signaling in HBECs is TRPA1-mediated, Ca^2+^-dependent, and ROS-sensitive. (a–c) Cells were exposed to medium alone or to 3% CSE for 6, 6, and 12 h, respectively, and pretreated with EGTA (an extracellular Ca^2+^ chelator; 500 *μ*M), HC-030031 (HC, a TRPA1 antagonist; 9 *μ*M), or N-acetyl-cysteine (NAC, a ROS scavenger; 1 mM). Protein expression was analyzed by Western blotting. Activation of ERK and JNK was indicated by increased phosphorylation of these kinases, whereas activation of NF-*κ*B was indicated by the increased presence of p65 subunit in the nucleus. Data in each group are mean ± SEM from five independent experiments. ^∗^ *p* < 0.05 versus the control; ^#^ *p* < 0.05 versus CSE alone.

The activation of ERK, JNK, and NF-*κ*B is known to be a signaling pathway that is central to the induction of IL-8 by CSE in HBECs [3, 5–8]. Exposure to CSE was able to increase the presence of the phosphorylated ERK (Figure 5(a)) and phosphorylated JNK (Figure 5(b)) in the cytosol and of the p65 subunit of NF-*κ*B in the nucleus (Figure 5(c)). Such CSE-induced activation of the MAPKs/NF-*κ*B signaling was significantly attenuated by pretreatment with EGTA (500 *μ*M), by pretreatment with HC030031 (9 *μ*M), and by pretreatment with N-acetyl-cysteine (Figure 5; 1 mM).

### 3.6. CS Increases the Expression of TRPA1 in Lung Epithelium and Lung Tissues of Mice

**Figure 6:**
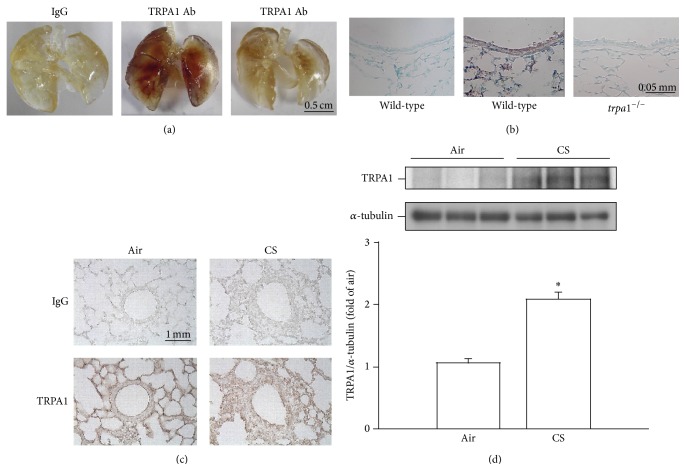
Cigarette smoke (CS) increases the lung expression of TRPA1 in mice. Mice were chronically exposed to air or CS for 4 weeks. (a) Representative images showing* en face* immunostaining with a TRPA1 antibody using whole lungs obtained from air-exposed wild-type mice and from* trpa1* ^−/−^ mice. (b) Representative images showing lung section obtained from the lungs with the* en face* TRPA1-immunostaining of air-exposed wild-type mice and* trpa1* ^−/−^ mice. (c) Representative images showing immunostaining with a TRPA1 antibody of representative lung sections obtained from air-exposed and CS-exposed wild-type mice. The specificity of the immunostaining was confirmed using an IgG-negative control. (d) Expression levels of TRPA1 in lung tissues were obtained from air-exposed and CS-exposed wild-type mice and were analyzed by Western blotting. ^∗^ *p* < 0.05 versus the air control group in both genotypes. Data in each group are mean ± SEM from nine mice.

In air-exposure animals,* en face* immunostaining showed strong signals across the whole of the lungs of wild-type mice (Figure 6(a), middle panel), but such signals were relatively weak when similarly treated* trpa1* ^−/−^ mice were examined (Figure 6(a), right panel). These results suggest that the geometry of the airways could be clearly visualized in treated wild-type mice, but not in treated* trpa1* ^−/−^ mice. Analysis of wild-type mouse lung sections obtained from lungs that had undergone* en face* immunostaining indicated that their lung epithelial cells (Figure 6(b), middle panel) displayed much stronger positive staining for TRPA1 than lung epithelial cells from* trpa1* ^−/−^ mice (Figure 6(b), right panel); these results indicate a difference in the expression levels of TRPA1 in the lung epithelium from the two types of mice. Additionally, immunohistochemical analysis showed that there was stronger positive staining for TRPA1 in the epithelial cells of lung sections from wild-type mice that had been chronically exposed to CS for 4 weeks compared to the air-exposure control mice (Figure 6(c)). The increase in relative expression level of TRPA1 measured as positive cells by immunostaining with the CS-exposure group was 2.1 ± 0.1-fold (*n* = 9), which is significantly higher than that found for the air-exposure group (1.0 ± 0.1-fold; *n* = 9). Furthermore, Western blot analysis revealed that expression of TRPA1 in the lung tissues of CS-exposure wild-type mice was also significantly higher than that of air-exposure control mice (Figure 6(d)).

### 3.7. CS-Induced Oxidative Stress and Lung Inflammation Are Lessened in* trpa1* ^−/−^ Mice

**Figure 7:**
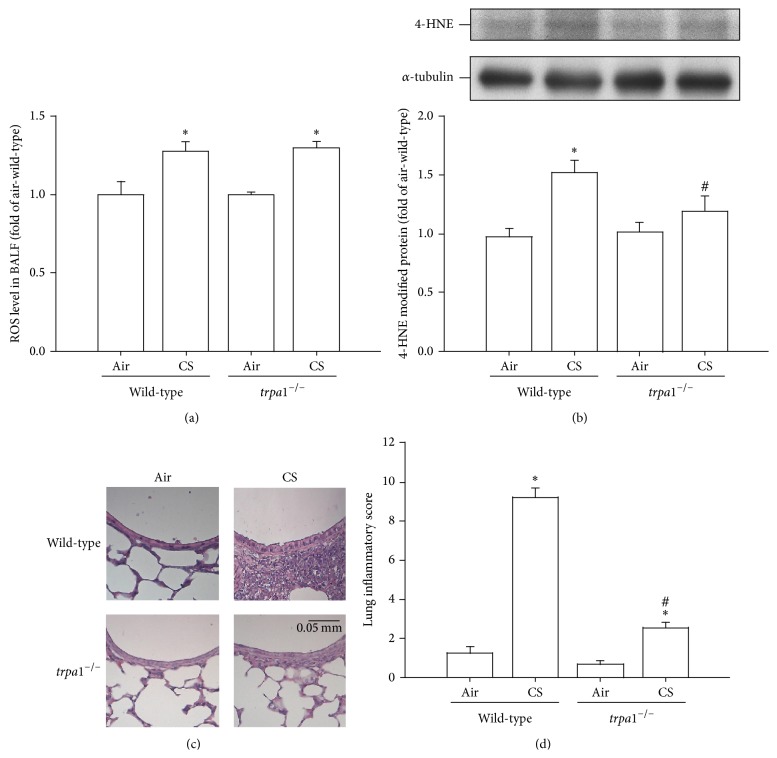
Comparisons of cigarette smoke- (CS-) induced oxidative stress and lung inflammatory score in wild-type and* trpa1* ^−/−^ mice. Mice were chronically exposed to air or CS for 4 weeks. (a) Levels of ROS in the bronchoalveolar lavage fluid (BALF) were sampled immediately after the last air or CS exposure across the four study groups. Levels of ROS were measured by DCFH-DA fluorescent probe assay. (b) Expression of 4-HNE modified protein, a biomarker of oxidative stress, in lung tissues was analyzed by Western blotting. (c) Representative images of H&E stained lung sections obtained from air-exposure or CS-exposure wild-type and* trpa1* ^−/−^ mice. (d) Lung inflammatory scores were calculated according to the sum of the levels of cell infiltration and damage levels assessed from the lung sections. Data in each group are mean ± SEM from nine mice. ^∗^ *p* < 0.05 versus the air-exposure group in both genotypes; ^#^ *p* < 0.05 versus the CS-exposure wild-type group.

**Figure 8:**
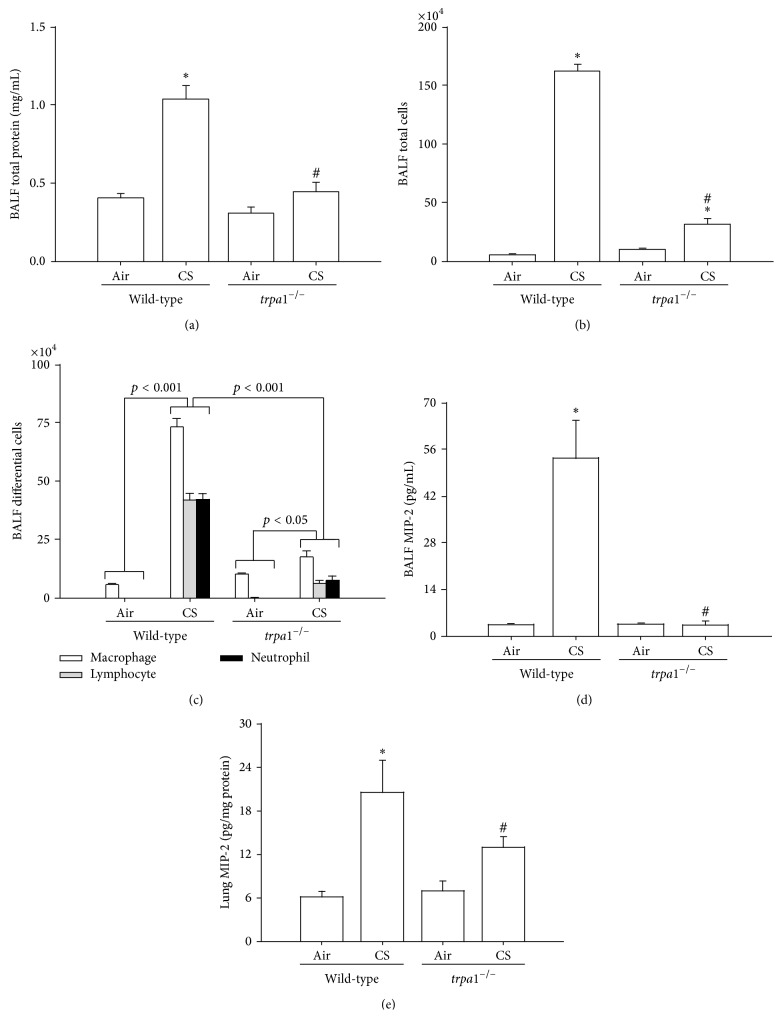
The cigarette smoke- (CS-) induced lung inflammation is alleviated in TRPA1 knockout mice. Mice were chronically exposed to air or CS for 4 weeks. Total protein content (a), total cell count (b), and differential cell count (c) obtained from the bronchoalveolar lavage fluid (BALF) were measured and served as indications of lung inflammation. (d, e) Levels of MIP-2 in BALF and in lung tissues were analyzed by ELISA. Data in each group are mean ± SEM from nine mice. ^∗^ *p* < 0.05 versus the air-exposure group in both genotypes; ^#^ *p* < 0.05 versus the CS-exposure wild-type group.

Analysis was carried out using a fluorescent probe and the results indicated that the level of ROS in the BALF, which is able to directly stimulate the airway epithelium, was increased in mice after CS exposure for 4 weeks compared to the same measurements for air-exposure mice (Figure 7(a)). However, no significant difference in the BALF levels of ROS was found between the CS-exposed wild-type and the* trpa1* ^−/−^ mice under the same conditions (Figure 7(a)). By way of contrast, Western blot analysis revealed that wild-type mice after exposure to CS showed increased levels of 4-HNE modified proteins (a product of lipid peroxidation) in their lungs compared to air-exposure wild-type mice (Figure 7(b)). This CS-induced increase in the level of 4-HNE, however, was greatly attenuated in the lungs of CS-exposure* trpa1* ^−/−^ mice relative to the wild-type mice (Figure 7(b)). A histological evaluation of the lung sections of CS-exposure wild-type mice revealed extensive infiltration of inflammatory cells, thickening of the alveolar walls, and the presence of abnormal reepithelialization and all of these changes were found to be less in the CS-exposure* trpa1* ^−/−^ mice (Figure 7(c)). The difference in the degree of lung inflammation between the CS-exposure wild-type and* trpa1* ^−/−^ mice was confirmed by comparing the group data in terms of lung inflammatory scores (Figure 7(d)). Furthermore, relative to the air-exposure wild-type mice, wild-type mice with CS exposure were found to show increases in BALF for total protein levels (Figure 8(a)), for total cell counts (Figure 8(b)), for differential cell counts (Figure 8(c)), and for MIP-2 levels (Figure 8(d)) as well as an increase in the level of MIP-2 in lung tissues samples (Figure 8(e)). All of these inflammatory indices were significantly lower in* trpa1* ^−/−^ mice exposed to CS (Figure 8).

## 4. Discussion

**Figure 9:**
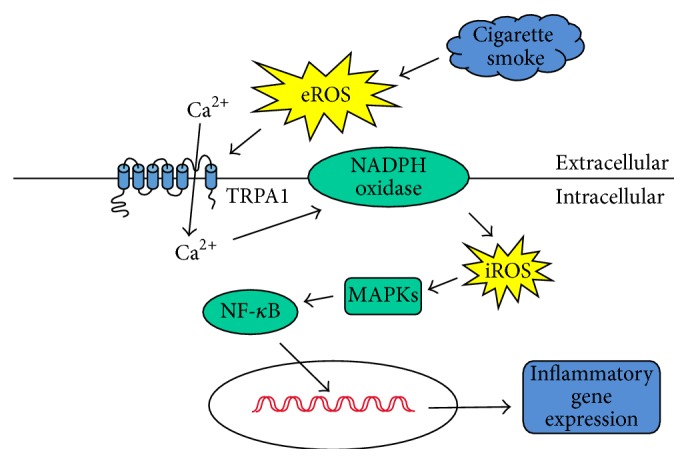
The proposed mechanism of TRPA1 activation for the induction of IL-8 by cigarette smoke (CS). As shown, exposure to CS initially causes an increase in the extracellular level of reactive oxygen species (ROS), which in turn activates lung epithelial TRPA1; this leads to the promotion of a Ca^2+^ influx. The increase in the intracellular level of Ca^2+^ in lung epithelial cells then contributes to the activation of NADPH oxidase, which in turn results in an elevation of the intracellular level of ROS and this then activates the mitogen-activated protein kinases (MAPKs)/nuclear factor-*κ*B (NF-*κ*B) signaling pathway allowing induction of IL-8.

Our* in vitro* study confirms the important role of lung epithelial TRPA1 in the induction of IL-8 by CSE in HBECs [21, 22]. We then used this* in vitro* model to investigate how lung epithelial TRPA1 acts as a crucial regulator during the transcriptional regulation of IL-8 induction by CSE. We found that CSE sequentially caused increases in extracellular ROS, intracellular Ca^2+^ level via ion influx, NADPH oxidase activity, and intracellular ROS. CSE then activated the MAPKs/NF-*κ*B signaling and induced IL-8 in HBECs. The time courses of these events are similar to those reported previously [6, 7, 21, 22]. Using various experimental interventions, we have identified the cascade of these events (Figure 9). Initially, exposure to CSE causes an increase in the extracellular level of ROS, which in turn activates lung epithelial TRPA1; this leads to the promotion of a Ca^2+^ influx. The increase in the intracellular level of Ca^2+^ of the HBECs then contributes to the activation of NADPH oxidase, which in turn results in an elevation of the intracellular level of ROS. Finally, this activates the MAPKs/NF-*κ*B signaling pathway, which brings about the induction of IL-8. These observations suggest that lung epithelial TRPA1 may transduce the presence of extracellular ROS induced by CS into the transcriptional regulation of lung inflammation via an influx of Ca^2+^.

Exposure of CS or CSE has been shown to increase ROS in extracellular fluid [7, 9] and in BALF [7, 10]. In this study, the increase in extracellular ROS was evoked by CSE as early as 2 min after exposure; however, at this time point the level of intracellular ROS remained unaltered. Although CS-induced extracellular ROS are known to insult the lung epithelium, which leads to lipid peroxidation and damage of cell membranes [30], its role in the transcriptional regulation of CS-induced lung inflammation has remained largely unclear up to the present. By way of contrast, a CS-induced increase in intracellular ROS has been widely accepted as serving to trigger the activation of ROS-sensitive inflammatory signaling in lung epithelial cells [6, 7] and in other types of lung cells [11–14]. Our findings regarding the activation of TRPA1 by extracellular ROS are not totally surprising because this event has already been demonstrated for neuronal TRPA1 [16, 31, 32] and in pancreatic beta cells [33], as well as in cells that have been transfected with TRPA1 [16, 31, 34]. Since ROS is strongly electrophilic, ROS may cause the oxidation of disulfide bonds near the pore region of these channels [35] or bring about the covalent modification of cysteines within the electrophile/oxidant-sensing site, which consists of three cysteines and one lysine found at N-terminus of this type of channel [16, 31, 35, 36]; either or both of these changes may lead to activation of TRPA1. Similar ROS related regulation of other types of TRP channels has been reported elsewhere [37].

In this study, the CSE-induced and TRPA1-mediated increase in intracellular Ca^2+^ seems to form a Ca^2+^ signaling event that activates the MAPKs/NF-*κ*B signaling pathway, which then results in the induction of IL-8. One event evidently triggered by this Ca^2+^ signaling is activation of NADPH oxidase. This requires the translocation of the p47^phox^ subunit from the cytosol to the membrane compartment, which was demonstrated in two of our previous studies [6, 7]. Indeed, an elevation of the intracellular Ca^2+^ concentration is known to act as an upstream signal for the activation of NADPH oxidase during cellular stress [38, 39]. To this end, many studies have reported that activation of NADPH oxidase is responsible for the CS-induced increase in intracellular ROS in lung cells [6, 7, 11–14]. Thus, lung epithelial TRPA1 provides an important link between the initial increase in extracellular ROS and the subsequent increase in intracellular ROS, both of which occur in response to CS exposure. In vascular smooth muscle cells, an interplay between intracellular Ca^2+^ and ROS has been suggested; furthermore, the Ca^2+^ signaling mediated by various Ca^2+^ channels has been shown to regulate the activity of NADPH oxidase in these cells and to increase intracellular ROS reciprocally via the activity of these Ca^2+^ channels [39]. Perhaps, this interplay can explain why, after the initial surge of Ca^2+^ influx, the intracellular Ca^2+^ remains persistently elevated until the end of the observation period (30 min) when HBECs have been exposed to CSE. At this time, the transient increase in extracellular ROS has vanished, but nevertheless the increase in intracellular ROS remains. We, however, cannot exclude the possibility that the TRPA1-mediated influx of Ca^2+^ also serves as a Ca^2+^ signal that is able to activate downstream inflammatory signaling. For example, an increase in intracellular Ca^2+^ is known to be able to activate the MAPKs/NF-*κ*B pathway in human lung epithelial cells and bring about the release of IL-8 [40]. In this context, we have previously shown that activation of the ROS-sensitive MAPKs/NF-*κ*B pathway is important for induction of IL-8 by CSE in HBECs [6, 7]. The present study further characterizes this pathway and shows it is a process that depends on having a functional lung epithelial TRPA1 and the presence of an influx of Ca^2+^. This provides additional evidence that helps our understanding of the early events that are involved in the transcription regulation of IL-8 by CSE in HBECs.

To investigate the role of lung epithelial TRPA1* in vivo*, we employed an established murine lung inflammation model involving induction by chronic CS exposure for 4 weeks [6, 7, 23]. TRPA1 antibody was then infused into the airways to bring about* en face* immunostaining, which resulted in a clear visualization of the airway geometry in wild-type mice. This showed that there is expression of TRPA1 in lung epithelium; this finding was also supported by immunohistochemical analysis of lung sections obtained from the lungs with* en face* immunostaining. We additionally showed that chronic CS exposure was able to increase the protein expression of TRPA1 in lung epithelium and in lung tissue in general, a result that is consistent with our* in vitro* findings. The mechanism underlying this increase in protein expression of TRPA1 is not clear, but it would seem to involve transcriptional regulation because the mRNA level of TRPA1 is also increased. These observations imply that the inflammatory function of lung epithelial TRPA1 may be augmented in response to CS exposure via this increased expression. We have further demonstrated that, as compared to the wild-type mice,* trpa1* ^−/−^ mice displayed a lower level of CS-induced lung inflammation, which was identified based on an alleviation of increased vascular permeability, a reduction in inflammatory cell infiltration, and a decrease in inflammatory cytokine levels. Lung epithelium is a target for direct insult by CS and these cells play a vital role in the initiation and progression of CS-induced lung inflammation [2–4, 6, 7]; therefore we believe that the reduced lung inflammation observed in our* trpa1* ^−/−^ mice is, at least in part, due to a lack of epithelial TRPA1 in the* trpa1* ^−/−^ mice. Additionally, TRPA1 is expressed in primary sensory neurons, endothelial cells, macrophages, smooth muscle cells, and fibroblasts [17, 21, 22, 41–43], all of which may participate in the development of CS-induced lung inflammation [1, 2, 16, 22, 41]. Accordingly, the lack of TRPA1 in these cells may also contribute to the reduction in lung inflammation that was observed in our* trpa1* ^−/−^ mice. In this study, one intriguing finding is that the ROS levels in the BALF of our* trpa1* ^−/−^ mice were not different when CS-exposure wild-type mice and the* trpa1* ^−/−^ mice were compared, whereas the level of 4-HNE modified proteins in lung tissues was lower in the CS-exposure* trpa1* ^−/−^ mice compared to the wild-type mice. Since the BALF was sampled immediately after the last CS exposure, we speculate that the ROS level in BALF was directly related to the amount of CS inhaled, which indicates that the insults to the airway epithelium by ROS in BALF are similar in these two genotypes of mice. By way of contrast, the level of 4-HNE modified protein would seem to reflect the degree of lipid peroxidation caused by the total ROS present in the lung tissues [27]. This would additionally encompass the ROS generated by the infiltrated inflammatory cells such as macrophages and neutrophils [2]. Since infiltration of these inflammatory cells into the lung is alleviated in the CS-exposure* trpa1* ^−/−^ mice, it is reasonable to observe that there is a reduction in lung oxidative stress of the CS-exposure* trpa1* ^−/−^ mice compared to the wild-type mice.

In summary, our* in vitro* findings suggest that exposure to CSE initially causes an increase in the extracellular level of ROS, which in turn activates lung epithelial TRPA1. TRPA1 then transduces this stimulation induced by CS into the transcriptional regulation of lung inflammation via an influx of Ca^2+^ (Figure 9). Our* in vivo* findings indicate that, while the insults to the airway epithelium caused by the presence of ROS in the BALF after CS exposure are similar for both wild-type and* trpa1* ^−/−^ mice, the level of CS-induced lung inflammation is reduced in* trpa1* ^−/−^ mice compared to wild-type mice. Thus, a cellular mechanism may possibly be at work in mice chronically exposed to CS. Our findings provide significant novel information that will allow a better understanding of the pathogenic mechanisms associated with CS-induced lung inflammation and should help the development of potential therapies.
